# Ocular Refraction at Birth and Its Development During the First Year of Life in a Large Cohort of Babies in a Single Center in Northern Italy

**DOI:** 10.3389/fped.2019.00539

**Published:** 2020-01-29

**Authors:** Francesco Semeraro, Eliana Forbice, Giuseppe Nascimbeni, Salvatore Cillino, Vincenza Maria Elena Bonfiglio, Maria Elena Filippelli, Silvia Bartollino, Ciro Costagliola

**Affiliations:** ^1^Department of Medical and Surgical Specialties, Radiological Sciences, and Public Health, University of Brescia, Brescia, Italy; ^2^Department of Ophthalmology, University of Catania, Catania, Italy; ^3^Department of Experimental Biomedicine and Clinical Neurosciences, University of Palermo, Palermo, Italy; ^4^Department of Medicine and Health Sciences V. Tiberio, University of Molise, Campobasso, Italy

**Keywords:** physiological refraction, amblyopia, newborns, refractive screening, preterm babies

## Abstract

The purpose of this study was to investigate refraction at birth and during the first year of life in a large cohort of babies born in a single center in Northern Italy. We also aimed to analyze refractive errors in relation to the gestational age at birth. An observational ophthalmological assessment was performed within 24 h of birth on 12,427 newborns. Refraction was examined using streak retinoscopy after the administration of tropicamide (1%). Values in the range of between +0.50 ≤ D ≤ +4.00 were defined as physiological refraction at birth. Newborns with refraction values outside of the physiological range were followed up during the first year of life. Comparative analyses were conducted in a subgroup of babies with known gestational ages. The following distribution of refraction at birth was recorded: 88.03% of the babies had physiological refraction, 5.03% had moderate hyperopia, 2.14% had severe hyperopia, 3.4%, had emmetropia, 0.45%, had myopia, 0.94% had astigmatism, and 0.01% had anisometropia. By the end of the first year of life, we observed reductions in hyperopia and astigmatism, and stabilization of myopia. Preterm babies had a four-fold higher risk of congenital myopia and a three-fold higher risk of congenital emmetropia as compared to term babies. Refraction profiles obtained at birth changed during the first year of life, leading to a normalization of the refraction values. Gestational age at birth affected the incidence of refractive errors and amblyopia.

## What is Known

- Premature infants have a higher chance of developing myopia.
- Hyperopia decreases as the length of the eye increases.
- Astigmatism resolves spontaneously in almost all cases in the first year of life.

## What is New

- Anisometropia is present in a very limited number of cases.
- Ophthalmic screening is particularly indicated for preterm babies to identify potentially eye pathologies.
- Gestational age at birth affects the incidence of refractive errors and amblyopia.

## Introduction

The eye is maximally functional and efficient in a state of emmetropia, which also involves slight hyperopia. Thus, the basic prerequisite for a good level of refraction and optimal visual functioning is regularity in ocular growth. However, there are convincing data showing that in the early developmental phase, good visual functioning plays a decisive role in establishing correct ocular growth (1). This developmental process, which occurs early in life and leads to refractive modulation, is called emmetropization (2–4). This is not a stable condition, as it represents a gradual and complex growth process that begins at birth and ends during the first few years of life (5, 6). Several studies have shown that emmetropia should not be evaluated at birth, but rather, that mild hyperopia is the natural state of refractive development in children, and that emmetropia during childhood carries the risk of subsequent progression to myopia (5).

Abnormal refraction is the main risk factor for the development of amblyopia, which is the most common cause of visual impairment in children. Amblyopia leads to strabismus and a reduction in binocular vision. In addition, it may limit future employment opportunities and increase risk for psychosocial problems. It is a widely held clinical belief that the risk of developing amblyopia and strabismus can be reduced if abnormal refraction is promptly identified and corrected (7–9).

A binocular disparity refers to the difference in the images of a single object seen by each eye, due the difference in angular position on the retina, and the inherent light disparity between each eye. This subtle disparity between retinal images is detected by the brain and provides important information about the depth structure of the world around us (10). The ability to obtain information about the 3-D structure of visual scenes by comparing information collected separately and simultaneously from different lines of sight is called stereopsis (11). The critical period for stereopsis development extends through late infancy and early childhood, and continues at least to the age of four to six. However, experiences that interfere with the development of binocular vision during infancy may severely disrupt the normal development of stereopsis (12). Assessment of these risk factors in relation to refractive screening should help to identify children who are most likely to benefit from early corrective and preventive treatment (13).

Considering the importance of refraction in the development of amblyopia, we assessed the distribution of refraction in newborns and followed up babies considered to be at risk for future refractive-related ocular abnormalities for a period of 1 year. Moreover, we investigated the associations between refractive errors, gestational age (GA), and weight at birth in a cohort of 12,427 newborns.

## Patients and Methods

A collaborative observational study was conducted by the Departments of Ophthalmology and Neonatology of the University of Brescia, Italy, over a 5-year period. Ophthalmologic screening was performed on all newborns within 24 h of birth. Patient screening included spontaneous ocular motility examination, adnexa and anterior segment inspection, indirect fundus ophthalmoscopy, and cycloplegic retinoscopy. All children received one drop of proparacaine in each eye followed by one drop of tropicamide (1%) and then a second drop of tropicamide 5 min later. Streak retinoscopy was performed in a dimly lit room light with streak retinoscopy at least 20 min after the second tropicamide drop. Residual accommodation was excluded by taking several readings for each infant to assess variability in retinoscopy reflex. Findings were recorded after obtaining readings with no variations, as suggested by Varughese et al. (14).

Hyperopia values between +0.5 D and +4.00 D were considered to be within physiological refraction at birth (15). Newborns whose refraction did not fall within this range were followed up during the first year of life as part of the protocol. Inclusion criteria for the follow-up program were as follows: moderate hyperopia (+4.00 ≤ D ≤ +6.00 D), high hyperopia (> +6.00 D), emmetropia (0 ≤ D ≤ +0.5), myopia (D < 0), astigmatism (cylinder ≥ +1.50 D), and anisometropia (>2 D discrepancy between the eyes). Refraction was classified as pathological or physiological, according to the mentioned classification (16, 17). Babies with evidence of congenital ocular disease (congenital cataract, congenital glaucoma, colobomas, retinopathy of prematurity) and/or systemic diseases associated with a risk of ocular involvement were excluded from the study.

Comparative analyses were conducted in a subgroup of babies with known gestational age (GA). Newborns were classified as preterm if the delivery occurred before the 38th week of GA.

SPSS statistical software (IBM SPSS Statistics 23, 2016) was used for data analysis. Chi-square (χ^2^) and Kruskal–Wallis tests were used for statistical evaluations. Statistical significance was set at a *p* < 0.05.

## Results

During the study period, 14,687 babies were born at the Department of Neonatology.

Of these, 12,427 (85%) were included in the study. The distribution of refraction at birth was as follows: 88.03% of the babies had physiological refraction, 5.03% had middle-high hyperopia, 2.14% had high hyperopia, 3.4% had emmetropia, 0.45% had congenital myopia, 0.94% had astigmatism (in all cases hyperopic), and 0.01% had anisometropia (Table 1).

**Table 1:** Number of children in each refractive category at birth.

**Refractive category**	**Number of children**
Physiologic hyperopia	10,940
Moderate hyperopia	625
Severe hyperopia	266
Emmetropia	422
Myopia	56
Astigmatism	117
Anisometropia	1
Total	12,427

The second outcome of our study was to investigate the association between the GA and refraction at birth. Data on the GA were collected for ~1 year in a subgroup of 3,600 newborns. Preterm infants constituted 11.3% of the babies with hyperopia (106 babies in a sample of 938 babies with physiological hyperopia), 30% of the babies with emmetropia, and 34% of the babies with myopia.

The mean GA of babies with physiological refraction was 39.33 ±1.29 weeks, whereas babies with emmetropia were 37.47 ± 1.85 weeks old. Compared with physiological refraction, emmetropia was significantly more common in preterm babies (χ^2^ = 70.1; *p* < 0.001, Kruskal–Wallis test). The odds ratio of congenital emmetropia in preterm babies was 3.31 (95%, 2.45–4.47) (Table 2). The mean GA of babies with myopia was 37.68 ± 1.98 weeks. Compared with physiological refraction, myopia at birth was significantly more common in preterm babies (χ^2^ = 24.6; *p* < 0.05, Kruskal–Wallis test). The odds ratio of congenital myopia in preterm babies was 4.03 (95%, 2.1–7.48) (Table 2).

**Table 2:** Distribution of myopia, emmetropia, hyperopia, and physiological hyperopia at birth in term and preterm babies.

	**Preterm (<38 WGA) (%)**		**Term (≥38 WGA) (%)**		**Total**
Myopia	19	3.68	37	1.20	56
Emmetropia	127	24.61	297	9.62	424
Hyperopia	264	51.16	1,920	62.22	2,184
Physiological refraction	106	20.54	832	26.96	938
Total	516	100	3,086	100	3,602

*Physiological refraction was measured only in a subgroup of 3,600 newborns. WGA, Weeks of gestational age*.

**Figure 1:**
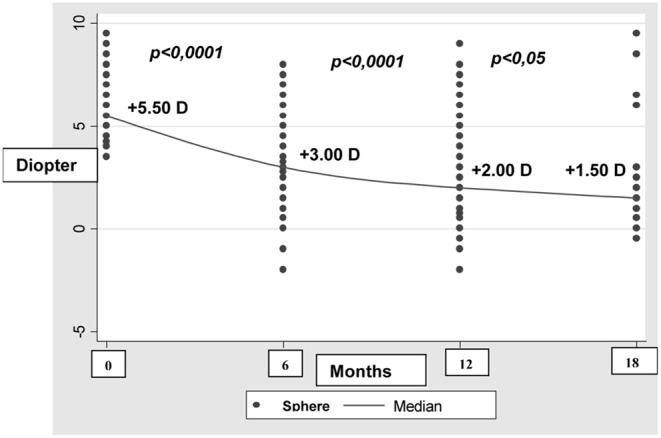
Trends in pathologic hyperopia during the first year of life.

We also analyzed the development of refractive errors during the first year of life (Tables 3 and 4). Table 3 shows data recorded from 915 out of 12,427 newborns during the first check 6 months after birth. The reduction in the sample size was due to participant drop-out. At the second check 12 months after birth, 447 out of 12,427 newborns were assessed (Table 4). During the 6 to 12 month follow-up period, hyperopia decreased as the length of the eye increased (Figure 1).

**Table 3:** Evolution of refraction in 915 babies evaluated 6 months after birth.

	**Physiologic hyperopia**	**Moderate hyperopia**	**Severe hyperopia**	**Emmetropia**	**Myopia**	**Astigmatism**	**Anisometropia**	
Physiologic hyperopia	0	0	0	0	0	0	0	0
Moderate hyperopia	20	263	91	0	1	9	1	385
Severe hyperopia	6	150	30	0	0	4	0	190
Emmetropia	51	79	1	56	24	6	1	218
Myopia	7	0	0	5	8	0	0	20
Astigmatism	11	39	6	6	5	35	0	102

**Table 4:** Evolution of refraction in 447 babies evaluated at 12 months after birth.

	**Physiologic hyperopia**	**Moderate hyperopia**	**Severe hyperopia**	**Emmetropia**	**Myopia**	**Astigmatism**	**Anisometropia**	
Physiologic hyperopia	0	0	0	0	0	0	0	0
Moderate hyperopia	185	3	0	4	1	2	0	195
Severe hyperopia	71	9	8	2	0	1	0	91
Emmetropia	50	18	0	23	5	5	0	101
Myopia	4	0	0	2	7	0	0	13
Astigmatism	37	1	0	1	1	7	0	47

*These results are separated from those reported in the table babies with anisometropia*.

**Figure 2:**
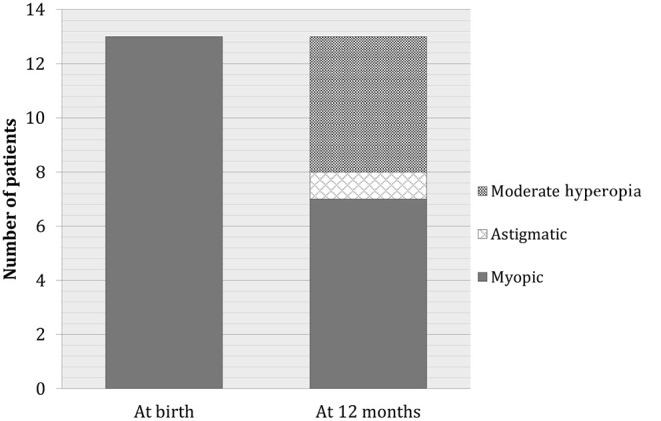
Evolution of myopia during follow-up.

Myopia developed in 5% of the babies with hyperopia, whereas no further worsening of myopia was recorded. At the end of the follow-up period, seven patients with myopia (50%) continued to have myopia, whereas six patients developed mild hyperopia or emmetropia. Moreover, six patients who previously had hyperopia or astigmatism developed myopia. At the end of follow-up, the total number of babies with myopia remained constant (Figure 2). Only seven of 47 babies (15%) remained astigmatic at the end of the first year, one baby (2%) reached emmetropization, another baby (2%) developed myopia, 38 babies (47%) developed hyperopia. Anisometropia disappeared in the first 6 months after birth. There were no significant differences in terms of sex distribution.

## Discussion

The aim of this study was to investigate the distribution of refraction at birth in a large cohort of babies and analyze its association with the GA. Changes in refraction during the first year of life in a subgroup of babies diagnosed with a pathological condition at birth were also evaluated.

Physiological hyperopia and non-physiological refraction were observed in 78% and 22% of the newborns, respectively. Refraction was classified as pathological or physiological according to the adopted classification: hyperopia up to +4.00 D was considered physiological at birth, as was hyperopia up to +3.50 D at 8 months (9) and up to + 2.00 D at 1 year. Myopia was defined by a threshold value of 0 D at birth, and −1.50 at 12 months (16–18). Babies with non-physiological refraction were grouped as detailed in Table 1. This included a considerable number of newborns with moderate to high hyperopia (625 with moderate hyperopia and 266 with severe hyperopia), a number of babies with hyperopia and astigmatism, a small number of babies with myopia, and one baby with anisometropia. This group of newborns was followed up over the subsequent year, to prevent further ocular diseases induced by uncorrected refractive errors. As expected, hyperopia decreased as the length of the eye increased, whereas newborns with myopic refraction remained essentially stable or improved during the follow-up period. This is consistent with previous findings showing that, during the first 2 years of life, common hyperopic refractive errors and the much rarer myopic errors rapidly reduce toward emmetropia.

A coordinated development in both corneal power and in axial length modifies the distribution of spherical equivalent refraction from an uniform to a highly peaked hyperopic distribution (3, 19). It has been established that refraction development is a complex process (20), and recent studies have shown that environmental changes seem to be responsible for the increase in myopia (21, 22). Refraction is not a simple trait, but is the result of an extensive range of processes that influence eye growth from gestation, through adulthood (6). The environment's influence on refractive developement is also supported by animal model studies showing that environmental factors can modulate the development of refraction (23–25). In other words, the process of growth in the first year of life leads to a reduction of myopia, but ultimately, environmental stimuli will determine adulthood refraction (21, 26–28). However, once myopia has developed, it can progress throughout childhood, leading to high myopia in adulthood (29).

In this study, astigmatism resolved spontaneously in the first year of life in almost all cases. This resolution occurs because of a physiological decrease in the toricity of the cornea and anterior lens, combined with a decrease in the variability of corneal lenticular surfaces. However, persistent infantile astigmatism is associated with increased astigmatism and myopia during school years (30–33). In this case, our data do not match those obtained by Miller et al. (34), who found that elevated higher-order aberrations to astigmatism and unsigned spherical aberration were minimal and not clinically significant in native American children. It is possible that this difference is due to ethnic differences between our study cohort from Italy and cohorts from other nations or regions.

Anisometropia was found in a very limited number of babies. It has been suggested that anisometropia at birth can resolve spontaneously over time. However, according to Almeder et al. (35) and to Deng et al. (36), the prevalence of anisometropia increases between 5 and 15 years, and thus, we cannot be exclude the possibility of some patients in this study developing anisometropia over time.

Accommodation in children is very powerful, and consequently, it is important to reach a healthly level of cycloplegia before retinoscopy. To reduce the risk of adverse reactions associated with atropine or cyclopentolate, we decided to use tropicamide (1%). However, as Mutti et al. found, when the drops are properly administered, the degree of difference between cyclopentolate and tropicamide is quite small, and there is a minimal effect on the measurement of distance refractive error and ocular optical components (37–39). We are confident that taking care in instilling drops, using of topical anesthetic, and allowing time before proceeding to streak retinoscopy were critical in achieving a suitable level of cycloplegia. Moreover, multiple readings were taken to verify the absence of variations due to residual accommodation (14). However, the possibility of a bias owing to incomplete cycloplegia cannot be excluded and could partially explain the low number of babies with astigmatism identified in our study.

Although cyclopentolate 0.5% is a better cycloplegic agent than tropicamide (1%) and has been used in other studies (40), it also produces systemic and local adverse events due to its muscarinic antagonist activity (41). For this reason, we chose to use tropicamide in our study. If any residual accommodation occurred, it would have resulted in a variability of the retinoscopy reflex, which we did not observe in this pilot study.

Other reasons responsible for the low number of anisometropia patients could be the use of a higher cut-off (cylinder ≥ +1.50 D, or > 2 D discrepancy between the eyes) values compared with other published studies.

Another interesting aspect of our study is the analysis of refractive errors in relation to GA in preterm and term babies. In particular, preterm babies were found to have a higher risk for congenital myopia and emmetropia, as compared to babies born at term. This is in agreement with previous studies (14, 42–44). The structural basis of preterm myopia has not been fully clarified, although some authors attributed it to a flat anterior chamber, increased corneal curvature, and spherical lens typical of the earlier phases of gestation.

In this study, we observed a reduction of hyperopia and astigmatism, and stabilization of myopia by the first year of life. Preterm babies had a four-fold higher risk of developing congenital myopia and a three-fold higher risk of developing congenital emmetropia, as compared to term babies.

We also found that refractive distribution obtained at birth changed during the first year of life, leading to a normalization of the refraction values, and that gestational age at birth affected the incidence of refractive errors and amblyopia. The strengths of this study was the large number of children analyzed. Its limitation was the lack of follow- up for babies with physiological hyperopia.

In conclusion, ophthalmic screening in preterm babies is primarily useful to identify potentially blinding pathologies (retinopathy of prematurity, congenital cataract, etc.), although this is difficult to diagnose in very young infants, particularly by non-eye doctors. The adoption of a screening protocol at birth is associated with a lower prevalence of amblyopia by scholar age, which allows sufficient time for early treatment of amblyopia (45).

Overall, the data collected in our study provide useful insight for understanding refraction in newborns.

## Data Availability Statement

All datasets generated for this study are included in the article/supplementary material.

## Ethics Statement

The study has been reviewed and approved by the Ethics Committee of Spedali Civili di Brescia. The study was performed in accordance with the principles of the Declaration of Helsinki and received the approval by the Institutional Review Board. Parental written consent was obtained for all participants, before inclusion in the study.

## Author Contributions

EF and GN performed data collection, writing, editing. SC, VB, MF, and SB contributed to data acquisition, review and editing, statistical analyses. FS and CC contributed to all facets of the work, and edited the manuscript.

### Conflict of Interest

The authors declare that the research was conducted in the absence of any commercial or financial relationships that could be construed as a potential conflict of interest.
